# Discovery of Plant Viruses From Tea Plant (*Camellia sinensis* (L.) O. Kuntze) by Metagenomic Sequencing

**DOI:** 10.3389/fmicb.2018.02175

**Published:** 2018-09-11

**Authors:** Xinyuan Hao, Weifu Zhang, Fumei Zhao, Ying Liu, Wenjun Qian, Yuchun Wang, Lu Wang, Jianming Zeng, Yajun Yang, Xinchao Wang

**Affiliations:** ^1^National Center for Tea Improvement, Tea Research Institute, Chinese Academy of Agricultural Sciences, Hangzhou, China; ^2^Key Laboratory of Tea Biology and Resources Utilization, Ministry of Agriculture, Hangzhou, China; ^3^Institute of Plant Protection, Henan Academy of Agricultural Sciences, Zhengzhou, China

**Keywords:** NGS, virus diversity, virus discovery, plant disease, leaf discoloration

## Abstract

The tea plant (*Camellia sinensis* (L.) O. Kuntze) is an economically important woody species. In this study, we collected 26 tea plant samples with typical discoloration symptoms from different tea gardens and performed metagenomic analysis based on next-generation sequencing. Homology annotation and PCR sequencing validation finally identified seven kinds of plant viruses from tea plant. Based on abundance distribution analysis, the two most abundant plant viruses were highlighted. Genetic characterization suggested that they are two novel virus species with relatively high homology to *Blueberry necrotic ring blotch virus* and *American plum line pattern virus*. We named the newly discovered viruses tea plant necrotic ring blotch virus (TPNRBV) and tea plant line pattern virus (TPLPV). Evolutionary relationship analysis indicated that TPNRBV and TPLPV should be grouped into the *Blunervirus* and the *Ilarvirus* genera, respectively. TPLPV might have same genome activation process with known ilarviruses based on sequence analysis. Moreover, specific primers for both viruses detection were designed and validated. The symptoms and ultrastructure of TPNRBV infected leaves were first recorded. Virus detections in the symptomatic and asymptomatic tissues from field plants showing tea plant necrotic ring blotch disease suggest that TPNRBV has a systemic movement feature. In summary, we first identified seven kinds of putative plant viruses by metagenomic analysis and report two novel viruses being latent pathogens to tea plant. The results will advance our understanding of tea plant virology and have significance for the genetic breeding of tea plants in the future.

## Introduction

The tea plant (*Camellia sinensis* (L.) O. Kuntze) is a perennial woody species first discovered as a drink and medicine in China approximately 3000 years ago. In addition to China, the tea plant has been widely cultivated in India, Sri Lanka, Kenya, Japan, and other countries. Tea production has great economic importance around the world because tea is second to water in terms of worldwide beverage consumption (Phipps, 1999; Khan and Mukhtar, 2008). For a long time, no viruses had been reported infecting tea plants, so it was thought that the tea plant was a virus-free species because that the catechins rich in tea leaves have anti-infective activities against a broad spectrum of viruses and other pathogens (Song, 2018). In recent years, metagenomics, based on the wide application of next-generation sequencing (NGS), has been frequently performed to investigate virus diversity and to discover novel viruses (Roossinck et al., 2015; Hadidi et al., 2016; Bernardo et al., 2017). As of 2016, over a thousand virus species had been discovered from wild and cultivated plants (Roossinck et al., 2015; Adams et al., 2016). However, up to date NGS had not been employed to search for viruses in tea plants.

Leaf discoloration, commonly including albino and chlorina symptoms, can be easily found in tea gardens, particularly in the autumn. Many leaf color cultivars have been widely cultivated in China. These cultivars usually produce higher free amino acid concentrations when young shoots are picked and generate tea products with better taste compared with regular green leaf cultivars (Li et al., 2011; Li C.F. et al., 2015; Wang et al., 2014). However, many tea plants with discoloration symptoms show weak growth status and even display significant symptoms of disease on certain conditions. The molecular mechanism leading to leaf color variation in the tea plant has been extensively studied but remains mostly unknown (Wang et al., 2016; Xu et al., 2017). Though discoloration symptoms might be caused by many factors, such as nutritional deficiency, pest or disease damage, genetic variation, and environmental stresses, virus infection is often considered as a possible cause (Chiumenti et al., 2015). Potentially, the disease caused by virus might be attributed to a fungal or bacterial agent based on the characteristic necrotic ring symptoms (Quito-Avila et al., 2013). Tea plants have a long lifespan bringing challenges to disease management. Usually, virus infection will cause serious damages to woody species (Pallás et al., 2012). Unfortunately, no study has been performed on virus identification in tea plants. Viruses can play multifaceted roles, including being a destroyer of agricultural products like the well-known tobacco mosaic virus (Koch et al., 2016), being a helper causing beneficial changes like the tulip breaking virus (Polder et al., 2010), and being a value tool in non-model-plant genome engineering (Xia et al., 2017). Therefore, the identification of plant viruses from the tea plant will be important to plant virology and tea plant breeding.

In this study, we collected tea plant samples with varied discoloration symptoms from different tea gardens in Hangzhou, China, and detected the diversity and transcript abundance of plant viruses using a metagenomic assay. Finally seven kinds of plant viruses were identified, and the two most abundant ones were characterized.

## Materials and Methods

### Plant Material Collection and Preparation

Twenty-six tea plant leaves or shoots with typical leaf discoloration (mainly includes albino and chlorina) symptoms were collected from different tea gardens in the fall of 2016 in Hangzhou, Zhejiang province, China (N 30°18′, E 120°10′). Total RNA from individual plant materials was isolated independently using the CTAB method described by Chang et al. (1993). Genomic DNA from the extracted total RNA was depleted by DNase I treatment (Invitrogen, Carlsbad, CA, United States). RNA quality was verified by 1% gel electrophoresis, and RNA concentration was quantified using a NanoDrop^®^ 2000c Spectrophotometer (Thermo Fisher Scientific, Waltham, MS, United States). The 26 RNAs were combined in equal amounts for RNA-Seq library construction. For PCR validation, 15 plant materials with leaf discoloration were randomly collected in the summer and fall of 2017 in Hangzhou. For systemic movement analysis, different tissues including apical buds, young stems, upper leaves (without visual symptoms), lower leaves (with reddish-brown rings or blotches), and roots from different diseased tea plants showing necrotic ring blotch symptoms were sampled in the summer of 2018 in Hangzhou, five biological replicates were carried out. Moreover, 15 individual shoots and leaves showing mixed symptoms of necrotic ring blotch disease were collected from different tea plants. RNA isolation and genomic DNA removal were performed as above.

### Library Construction and Sequencing

TruSeq^®^ Stranded Total RNA with Ribo-ZeroTM Gold Kit (Illumina, San Diego, CA, United States) was used to remove ribosomal RNA and construct the RNA-Seq library according to the manufacturer’s instructions. The final sequencing library was subjected to quality control using an Agilent 2100 Bioanalyzer (Agilent Technologies, Palo Alto, CA, United States). High-throughput RNA sequencing was performed on a HiSeq X Ten (Illumina) sequencer, and paired-end reads of 150 bp were obtained.

### Sequence Trimming, *de novo* Assembly, Annotation, and Abundance Quantification

Adapter sequences were first removed from harvested raw data using the Cutadapt (v1.15) program, and then low-quality and unpaired reads were trimmed using the Sickle quality-based trimming program^1^. The read quality was evaluated using the FastQC program. Non-virus sequences were filtered from clean data by the BBMap (v35.59) program^2^ with a mismatch rate of 3 bases/read on the basis of the available transcriptome (Submission number in NCBI: SRR5040773 to SRR5040784, and SRA061043) and genome data of the tea plant (Xia et al., 2017). Trinity (v2.2.0)^3^ was applied to the non-virus sequences, and the *de novo* assembly transcripts were evaluated by the Compute Contig Statistics program in the Discovery Environment of CyVerse^4^. By BLAST (v2.2.30+) analysis with the non-redundant protein sequence (nr) database, nucleotide collection (nt) database, tea genome database and virus genome database^5^, the best hits (with a significant *E*-value of <1e^-5^) in each database were assigned to the assembled transcripts of the tea plant. Viruses were classified according to the last virus taxonomy released by the International Committee on Taxonomy of Viruses (ICTV) in 2016. The abundance of each transcript was quantified using the RSEM (v1.2.11) program (Li and Dewey, 2011).

### cDNA Synthesis and Sequencing Validation of Annotated Virus Fragments

RNAs were reverse-transcribed into cDNAs using the SuperScript^®^ III First-Strand Synthesis System (Invitrogen, Carlsbad, CA, United States) according to the manufacturer’s instruction [oligo(dT) was replaced by the degenerate primer N25: 5′-NNNNNNNNNNNNNNNNNNNNNNNNN-3′]. The final products were diluted with 400 μL of ultrapure water for PCR validation. The top-abundant transcript of each annotated virus was used for sequencing validation. Primer pairs were designed by the online program Primer-BLAST^6^. For PCR, a 10 μL mixture containing 1 μL of cDNA template, 5 μL of 2× master mix (Roche, Indianapolis, IN, United States) and 0.5 μL of each primer (20 pmol) was set up. PCRs were performed with a program of 95°C for 10 min and 35 cycles of amplification with 95°C for 20 s, appropriate annealing temperature for 10 s, and 70°C for 35 s. Primer and annealing temperature information were provided in **Supplementary Material 1**. PCR products were monitored by 1.5% gel electrophoresis, and then the main bands were recovered by gel extraction and sequenced on Sanger platform.

### Full-Length Genome Sequence Cloning and Bioinformatics Analysis

Full-length genome sequences were cloned by rapid amplification of cDNA ends (RACE) using the SMARTer^®^ RACE 5′/3′ kit (TaKaRa, Kusatsu, Japan). A Marathon^®^ cDNA Amplification Kit (TaKaRa, Kusatsu, Japan) was used to amplify the middle, 5′ end, and 3′ end following the kit’s instructions. The primer information for full-length genome sequence cloning is presented in **Supplementary Material 2**. To confirm the presence of poly(A) tail at 3′ terminus, reverse transcription was redone using the SuperScript^®^ III First-Strand Synthesis System [oligo(dT) in kit was used], and then a forward specific primer together with oligo(dT) was used for the amplification of 3′ terminus fragment. Amplicon was purified and sequenced on Sanger platform. Sequence identity and similarity were detected by the EMBOSS Needle program, an online global alignment tool provided by EMBL-EBI^7^. Phylogenetic trees were inferred using the neighbor-joining method conducted in MEGA7.0 (Kumar et al., 2016) with 1,000 bootstrap replicates. Nucleic acid or protein sequences were aligned by ClustalW in MEGA7.0, and then edited on GeneDoc v2.7. RNA secondary structure predictions were performed using RNAcofold web server^8^ (Lorenz et al., 2011).

### Specific Primer Selection, RT-PCR Analysis, and Transmission Electron Microscopic (TEM) Detection

On the basis of full-length genome sequence information of TPNRBV and TPLPV, multiple primer pairs were designed by Primer-BLAST for specific primer selection. cDNAs from all the collected samples were mixed equally and then used as template in the selection. PCR and gel electrophoresis were performed following above descriptions. To detect the systemic movement characteristic of TPNRBV, different tissues, shoots, and leaves collected from diseased plants were monitored using the four specific primer pairs by reverse-transcription (RT)-PCR. RNAs from these tissues were independently reverse-transcribed into cDNAs using the SuperScript^®^ III First-Strand Synthesis System [oligo(dT) in kit was used]. PCR reactions were set up using KOD-Plus-Neo system (TOYOBO, Osaka, Japan) following the kit’s instructions, and 35 cycles of amplification were carried out. Finally PCR products were monitored by 2% gel electrophoresis. The PCR positive tissues with typical disease spot together with healthy control were separately cut into small pieces and fixed with 2.5% glutaraldehyde overnight at 4°C. The methods for sample preparation, ultrathin section, and electron microscopic examination were carried out following the descriptions by Wang et al. (2014).

## Results

### Sample Collection and Metagenomic Sequencing

**FIGURE 1:**
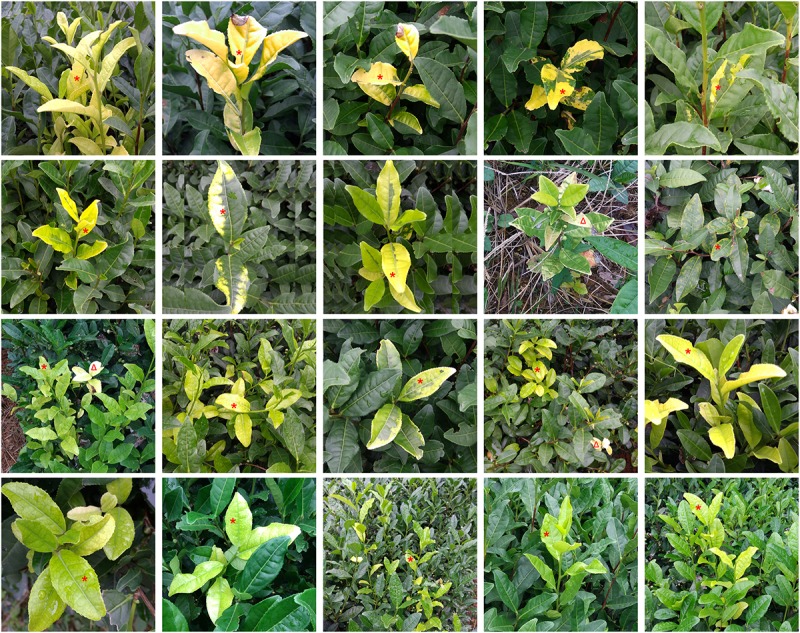
Representative discoloration symptoms among collected tea samples for metagenomic analysis; red asterisk (^∗^) indicates the chlorina leaves, red triangle (Δ) indicates the albino leaves.

In this study, 26 samples showing leaf discoloration were collected from tea gardens located in different regions of Hangzhou, China. All the samples were chosen for metagenomic sequencing. Partial samples with typical symptoms are shown in **Figure 1**, including the albino or chlorina phenomenon in one or two shoots or leaves from healthy, growing tea plants. A total of 108 Gbp of raw data in 643,055,788 paired-end reads were yielded after sequencing (SRA accession number in NCBI: SRP128758), and 102 Gbp of clean data were harvested after quality control and trimming. Finally, after removal of non-virus sequences, only 3 Gbp of sequences, 2.82% of the original sequencing data, were reserved (**Table 1**). The *de novo* assembly produced 142,773 contigs with N50 of 365 bp, and the longest contig was 13,897 bp.

**Table 1:** Statistical information of the sequencing data with or without trimming.

	Raw data	Clean data		
		Original	Removal of tea transcripts	Removal of tea transcripts and genome
Paired-end reads number (size)	643,055,788 (108 Gbp)	617,757,068 (102 Gbp)	463,883,124 (77 Gbp)	18,165,112 (3 Gbp)
Ratio to raw data	100%	96.07%	72.14%	2.82%

### Virus Identification and Abundance Distribution Analysis

**FIGURE 2:**
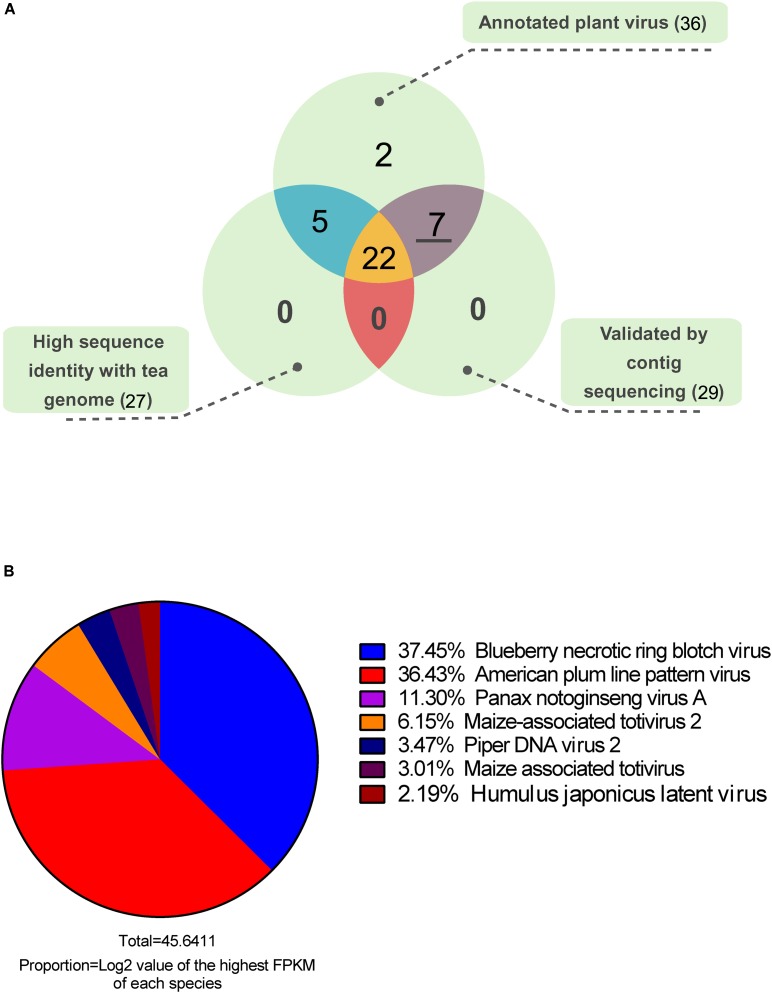
Venn diagram **(A)** of the annotated plant viruses and abundance distribution **(B)** of candidate viruses.

After functional annotation, 80.17% of the contigs were assigned to at least one of the nt, nr, and virus genome databases. Only 1.54% of the contigs were annotated as virus homologous sequences or virus protein-coding genes. Based on abundance quantification and functional annotation, the virus-annotated contigs were removed when their log2 (FPKM) values were lower than 1. Finally, 206 contigs from 36 plant virus species were identified as virus-associated sequences (**Supplementary Material 3**). To filter out false positives, we did sequence homology analysis between virus-annotated contigs and available tea genome sequence, and 27 virus species showed relative high sequence identity with tea genome, which were considered as putative insertion of virus related sequences in the plant genome (**Figure 2A**). Moreover, using the cDNA mixture from 26 collected samples as PCR template, the highest abundant contig of each annotated virus species were further validated by Sanger sequencing. More than one primer pairs were designed for the PCR if no productions or unique bands were obtained. Finally 29 out of 36 contigs were validated (**Supplementary Material 1**). Taking all into account, the seven plant viruses without homology with tea genome and validated by PCR amplification and sequencing were considered as real viruses existing in tea plants. Furthermore, an abundance distribution of the seven plant viruses was presented in **Figure 2B**. Among the viruses, blueberry necrotic ring blotch virus (BNRBV) and American plum line pattern virus (APLPV) took up the highest transcript abundance, followed by Panax notoginseng virus A (PnVA) and maize-associated totivirus 2 (MATV2), piper DNA virus 2 (PDV2), maize-associated totivirus (MATV), Humulus japonicus latent virus (HJLV).

### Discovery of Novel Viruses in Tea Plants

According to the homology annotation, five contigs were annotated as BNRBV protein-coding genes, and three as APLPV protein-coding genes (**Table 2**). PCR validation was performed to check the positive ones among 26 collected samples for the two most enriched plant viruses (**Supplementary Material 4**). The results indicated that 6 of 26 samples might have been infected by BNRBV or a related virus and that 4 of 26 samples might have been infected by APLPV or a related virus. Moreover, one sample might have been infected by both viruses. Based on this information, RACE was performed to obtain full-length genome sequences. After RT-PCR and Sanger sequencing confirmation, a complete genome sequence of 14,979 nt, comprising four segments (GenBank accession numbers: MG781152, MG781153, MG781154, and MG781155), and a complete genome sequence of 7,869 nt, comprising three segments (GenBank accession numbers: MG781156, MG781157, and MG781158), were obtained. Sequence analyses indicated that less than 60% identity and similarity were detected between the two cloned genome sequences compared with the corresponding BNRBV and APLPV sequences at both the nucleotide and amino acid levels (**Supplementary Material 5**). No sequence identity to other known viruses was detected by BLASTN search. These results suggest that two new virus species have been identified in the tea plant. Therefore, we tentatively named the virus that was similar to BNRBV as tea plant necrotic ring blotch virus (TPNRBV) and the virus similar to APLPV as tea plant line pattern virus (TPLPV).

**Table 2:** Identification of the two most abundant plant viruses in the tea plant.

Contig ID	Log2 of FPKM	Functional annotation				
		Putative coding protein	GenBank ID	Virus	*E*-value of homology	Identity %
TR61686| c0_g2_i1	15.04	Polymerase	YP_004901701.1	BNRBV	5.00E-150	34.75
TR91450| c2_g1_i1	4.47	Movement protein	AGI44303.1	BNRBV	8.00E-51	39.13
TR91450| c2_g1_i2	5.81	Movement protein	AGI44303.1	BNRBV	6.00E-49	39.13
TR46240| c4_g1_i1	17.09	Methyltransferase-helicase	AGI44297.1	BNRBV	2.00E-35	34.17
TR91450| c3_g1_i1	15.57	p24	YP_004901704.1	BNRBV	1.00E-14	35.96
TR32524| c6_g2_i1	16.41	Putative virus replicase p1	NP_602312.1	APLPV	0	41.81
TR32524| c6_g1_i1	15.75	Putative polymerase p2	NP_602313.1	APLPV	1.00E-166	53.62
TR32524| c6_g3_i1	16.63	Putative movement protein 3a	NP_602314.1	APLPV	1.00E-26	31.98

### Gene Structure and Sequence Analysis

**FIGURE 3:**
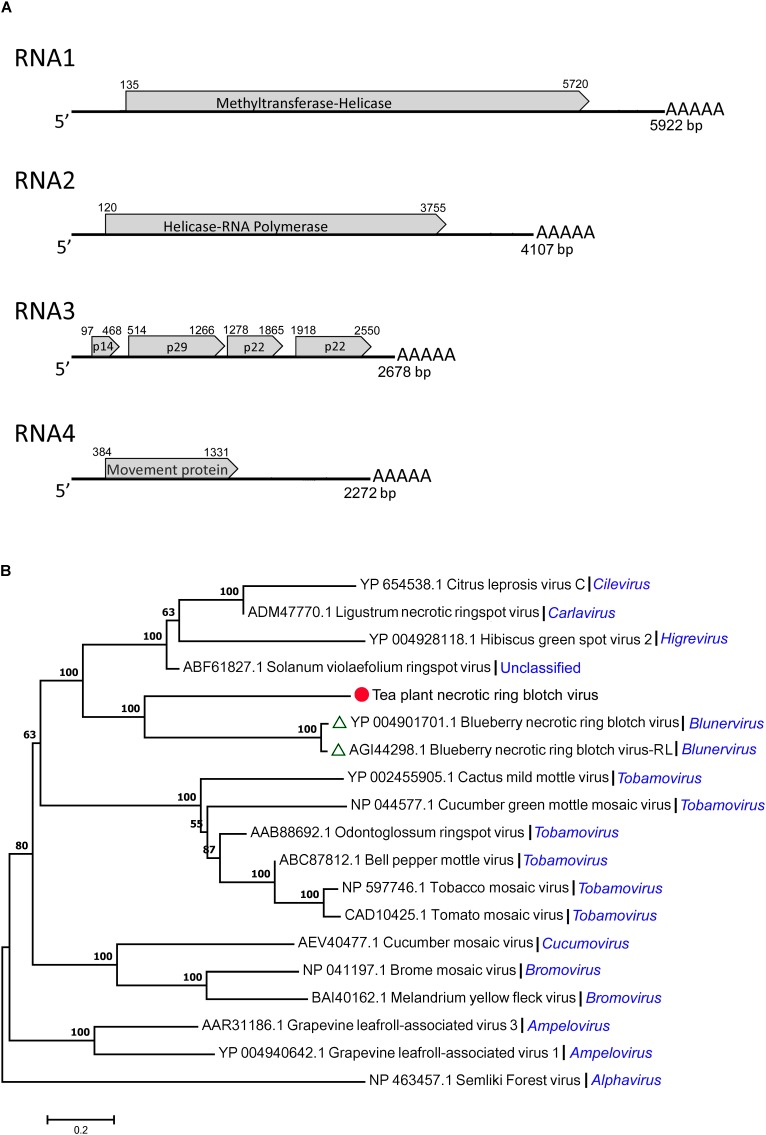
Genomic structure analysis **(A)** and phylogenetic tree analysis **(B)** of the tea plant necrotic ring blotch virus (TPNRBV). Graphic representation in **(A)** is not to scale. Poly(A) tails of varied lengths on the 3′ ends were represented by five A letters. Putative proteins are indicated according to their size and position in RNA3.

By gene structure analysis, the four segments (RNA1, RNA2, RNA3, and RNA4) of TPNRBV had poly(A) tails of varied lengths on the 3′ ends (**Figure 3A**). RNA1 was composed of 5922 nt and contained a long ORF spanning nucleotides 135 to 5720 and encoding a putative 212 kDa protein (1861 amino acids) having 22.8% identity with the methyltransferase-helicase encoded by BNRBV RNA1. Correspondingly, the RNA1 of TPNRBV encoding protein contained methyltransferase (MTR; aa 132–460), cysteine-protease (C-Pro; aa 691–828) and helicase (HEL; aa 1511–1802) domains like the RNA1 of BNRBV (Quito-Avila et al., 2013). RNA2 was composed of 4107 nt and contained a long ORF spanning nucleotides 120 to 3755 and encoding a putative 139 kDa protein (1211 amino acids) having 31.3% identity with the helicase-RNA polymerase encoded by BNRBV RNA2. Similarly, the RNA2 of TPNRBV encoding protein contained HEL (aa 263–566) at the N-terminus, and RNA-dependent RNA polymerase (RdRp; aa 759–1183) at the C-terminus like the RNA2 of BNRBV (Quito-Avila et al., 2013). RNA3 was composed of 2678 nt and contained 4 ORFs encoding 14 kDa (123 amino acids), 29 kDa (250 amino acids), 22 kDa (195 amino acids), and 22 kDa (210 amino acids) putative proteins; these proteins had only 10.6, 23.7, 26.5, and 25.5% identity, respectively, with the corresponding proteins encoded by RNA3 of BNRBV. RNA4 was composed of 2142 nt and contained one long ORF spanning nucleotides 384 to 1331 and encoding a 35 kDa (315 amino acids) putative movement protein. The movement protein contained a motif at position aa 65–250 conserved in the 30 K superfamily of virus movement proteins and had 37.3% identity with the protein encoded by RNA4 of BNRBV (Melcher, 2000; Quito-Avila et al., 2013). The non-coding regions (NCRs) ranged from 96 to 383 nt and from 128 to 941 nt at the 5′ and 3′ termini, respectively. No conserved sequence was found at the 5′ terminus of all segments. However, A-T content at the 3′ NCRs, including the homopolymeric tracts, was at very high level among the four segments (**Supplementary Material 6**). Moreover, the presence of a poly(A) tail was confirmed at each segment. A phylogenetic tree was constructed based on a putative RNA polymerase encoded by TPNRBV together with BNRBV, BNRBV-RL, and other viruses (**Figure 3B**). The results indicated that TPNRBV had the closest evolutionary relationship with BNRBV and BNTBR-RL (a strain of BNRBV). The phylogenetic analysis suggested that TPNRBV is a tentative new species in the genus *Blunervirus*.

**FIGURE 4:**
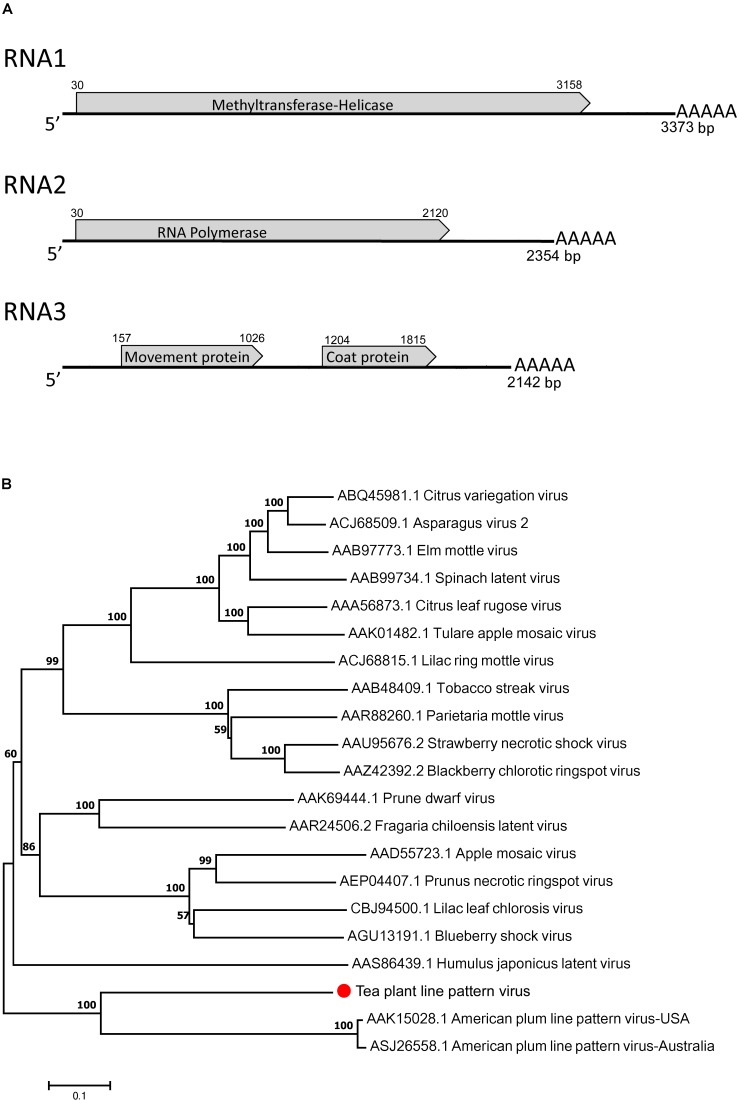
Genomic structure analysis **(A)** and phylogenetic tree analysis **(B)** of the tea plant line pattern virus (TPLPV). Graphic representation in A is not to scale. Poly(A) tails of varied lengths on the 3′ ends were represented by five A letters.

**FIGURE 5:**
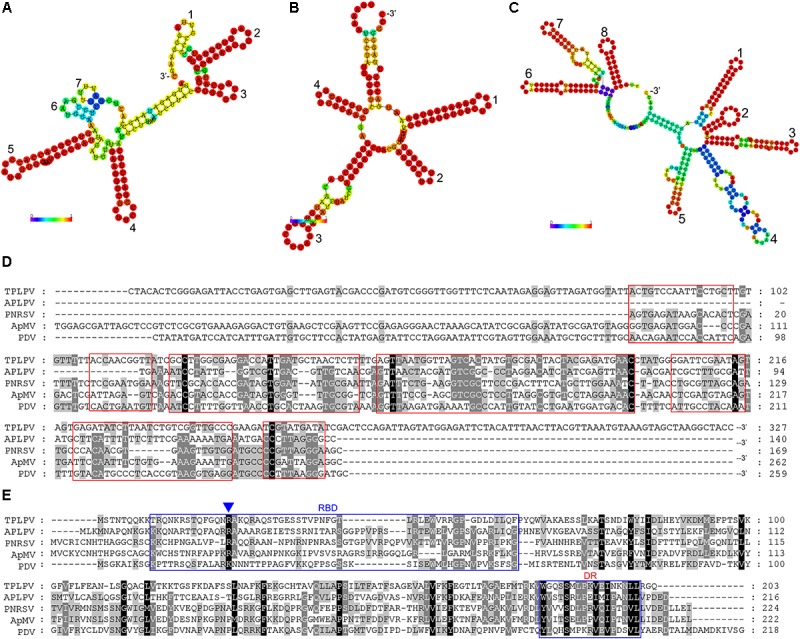
Secondary structure comparison **(A–C)**, alignment analysis of the 3′ untranslated region (3′UTR) of TPLPV **(D)** and alignment analysis of TPLPV coat protein **(E)** with related ilarviruses. **(A–C)**, Minimum free energy plain structure drawings of the 3′UTR of the PNRSV RNA3, American plum line pattern virus (APLPV) RNA3 and TPLPV RNA3, respectively. Stem-loop structures in each drawing are denoted from 3′ terminus (digits). Bars indicate the base pair probabilities. **(D)**, Alignment of the RNA3 3′UTR of TPLPV, APLPV, PNRSV, ApMV, and PDV. The red boxes indicate the corresponding regions of seven stem-loop structures in **(A)**. **(E)**, Alignment of the coat protein of TPLPV, APLPV, PNRSV, ApMV, and PDV. Putative RNA binding domain (RBD) and dimerization region (DR) in the coat proteins are indicated using blue boxes. The R residue in RBD was marked by a blue triangle (Δ), which is essential for the binding to the RNA.

The complete genome of TPLPV had three segments of 3373 nt (RNA1), 2354 nt (RNA2), and 2142 nt (RNA3) (**Figure 4A**). Like the TPNRBV, all the segments had a poly(A) tail of different lengths. RNA1 contained a long ORF spanning nucleotides 30 to 3158 and encoding a 117 kDa putative methyltransferase-helicase (1042 amino acids; MTR at aa 52–412; HEL at aa 717–1036) with 42.0% identity to the protein encoded by RNA1 of APLPV. RNA2 had a long ORF spanning nucleotides 30–2120 and encoding an 80 kDa putative RNA polymerase (696 amino acids; RdRp at aa 224–637) with 41.8% identity to the protein encoded by RNA2 of APLPV. RNA3 contained two large ORFs spanning nucleotides 157–1026 and 1204–1815. The putative protein located in the N-terminus had 289 amino acids (expected 32 kDa; contained a motif at position aa 43–267 conserved in the *Bromovirus* virus movement proteins), and the putative protein in the C-terminus had 203 amino acids (23 kDa; contained a motif at position aa 8–203 conserved in the *Ilarvirus* family virus coat proteins). These proteins had 24.8 and 31.7% identity, respectively, with the movement protein and coat protein encoded by RNA3 of APLPV. The evolutionary relationships of TPLPV with other viruses in the *Ilarvirus* genus were inferred using the neighbor-joining method based on the RNA polymerases encoded by RNA2 (**Figure 4B**). TPLPV had weak phylogenetic relationships with other members of the *Ilarvirus* genus. These results suggest that TPLPV is a new member of the *Ilarvirus* genus and could be classified into the same subgroup as *American plum line pattern virus*. Among TPLPV, APLPV, Prunus necrotic ringspot virus (PNRSV), apple mosaic virus (ApMV), and prune dwarf virus (PDV), alignment of the coat proteins indicated a high proportion of arginine (R) in N-terminus and a highly conserved region in C-terminus, which located at the same position with the RNA binding domain (RBD) and dimerization region (DR) of PNRSV coat protein, respectively (**Figure 5E**). Particularly, the residue 23 (R) of TPLPV coat protein highly conserved in the RBD. Secondary structure analysis further showed that eight stem-loop structures would form at the 3′UTR of TPLPV RNA3 (**Figure 5C**). Compared with the stem-loops in 3′UTR of PNRSV and APLPV, fewer stem-loops were observed in APLPV, and TPLPV had more similar stem-loop structures with PNRSV (**Figures 5A–C**). Furthermore, alignment of the TPLPV 3′UTR with APLPV, PNRSV ApMV, and PDV indicated a high conservation among them (**Figure 5D**).

### Specific Primer Pair Design and Virus Detection

**FIGURE 6:**
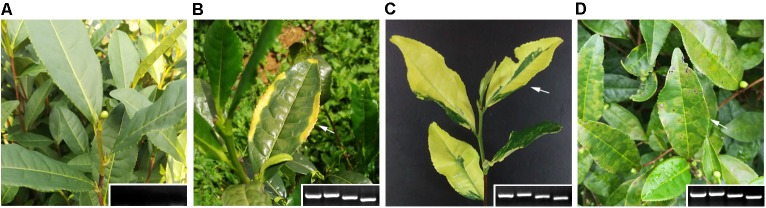
Virus detection by RT-PCR using specific primer pairs (**A**, negative control; **B–D**, TPNRBV-positive samples; the four bands at the lower right corner represent the PCR products of RNA1 to RNA4, respectively, in a 1% agarose gel).

**FIGURE 7:**
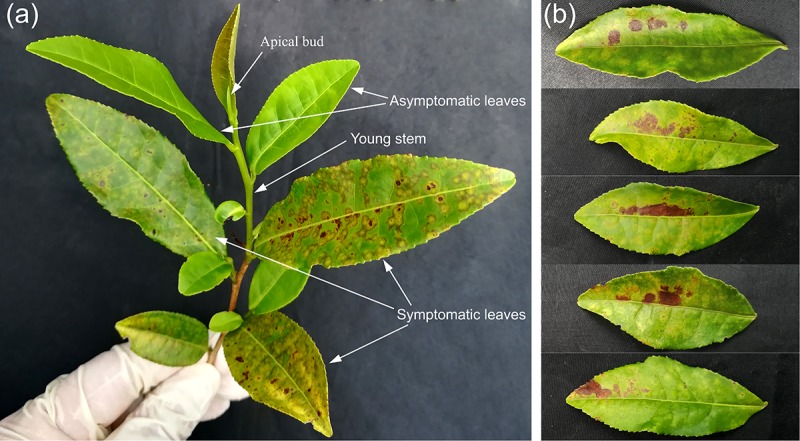
Typical appearances of tea shoot **(A)** and leaves **(B)** used in the systemic movement detection of TPNRBV by reverse-transcription (RT)-PCR analysis. **(A)**, A tea shoot showing mixed symptoms of tea plant necrotic ring blotch disease. **(B)**, Tea leaves in which one half of the leaf is symptomatic (upper) and the other half was not (lower).

**FIGURE 8:**
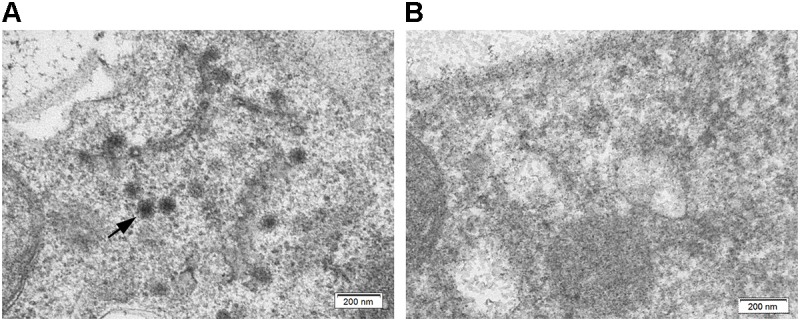
Ultrastructure of tea leaf infected by TPNRBV **(A)** and healthy control **(B)**; black arrow indicates the virus particles distributed in tea leaf. Scale bar, 200 nm.

Both TPNRBV and TPLPV had multiple segments. To accurately determine the presence of TPNRBV or TPLPV in tea plant, multiple primer pairs were designed and validated by PCR analysis using positive samples confirmed in **Supplementary Material 4**, and healthy mature tea leaves were used as a negative control (data not shown; **Figure 6A**). Finally, one specific primer pair was selected for each segment of the two viruses, and the sequence information is listed in **Table 3**. Using the specific primers, we detected the 15 samples randomly collected in Hangzhou in 2017. Three samples were TPNRBV infected, and no sample was infected by TPLPV. The symptoms of TPNRBV-infected leaves varied, including chlorina on the edge of the leaf (**Figure 6B**), mottled chlorina on a branch (**Figure 6C**), and multiple necrotic ring blotches on mature leaves (**Figure 6D**) distributed at the bottom of the tea plant. The necrotic ring blotch symptom can be easily observed in multiple tea cultivars in many tea gardens. Also, the infected plants show same symptom in the second year. Virus distribution studies showed that all the detected tissues including apical buds and roots were TPNRBV positive (**Table 4** and **Supplementary Material 7**). Furthermore, 15 individual shoots and leaves showing mixed symptoms of necrotic ring blotch disease were also TPNRBV positive either in symptomatic tissues or in asymptomatic tissues (**Table 5**, **Figure 7**, and **Supplementary Material 7**). Particularly, the diseased leaves with necrotic ring blotch symptom were sampled and observed using TEM. Compared with healthy control (**Figure 8B**), multiple virus particles of TPNRBV were detected in cytoplasm of infected-leaf tissue (**Figure 8A**). The particles were in spherical shape with a diameter approximately 85 nm.

**Table 3:** Specific primer information for tea plant necrotic ring blotch virus (TPNRBV) and tea plant line pattern virus (TPLPV) detection.

Virus segment	Primer name	Sequence (5′ to 3′)	Annealing temperature	Amplicon length
TPNRBV RNA1	TPNRBV1-F	GCCCTGACAACGCAAAAGAACTGATG	52°C	535 bp
	TPNRBV1-R	GTGACGGGATATTTTTGGACGACTGT		
TPNRBV RNA2	TPNRBV2-F	GGGCCGGGGTGTGGAAAAACTT	52°C	551 bp
	TPNRBV2-R	TTCTTATCATCCCGGCAAAACACA		
TPNRBV RNA3	TPNRBV3-F	TTCGCCACTCACAAAGACAACAAACT	52°C	459 bp
	TPNRBV3-R	GTAGCGGAGCGGAAAGAAAAGACT		
TPNRBV RNA4	TPNRBV4-F	TCAGTGGCGCGATTATCAGAAGGTA	52°C	407 bp
	TPNRBV4-R	CGCGCAAGAAGTCGGTCAAAAC		
TPLPV RNA1	TPLPV1-F	AAGGTGGCGAGGTCAGTTTCAGTTCA	52°C	513 bp
	TPLPV1-R	TCCCCATAGGTTCATCTTGTAGCAGTCG		
TPLPV RNA2	TPLPV2-F	CCTATGGAGCTCTATGACGCAAATGAT	52°C	403 bp
	TPLPV2-R	TCGACAGAGAAGTGATGGGGAAATAC		
TPLPV RNA3	TPLPV3-F	TCAGATGCGACCAATGGAACTCAACT	52°C	421 bp
	TPLPV3-R	CCCGCGCTAATCCTCTCAAGACTAACTA		

**Table 4:** Detection of TPNRBV via reverse-transcription (RT)-PCR in different tissues of tea plant displaying symptoms of tea plant necrotic ring blotch disease.

Number of biological replications^b^	Different tissues^a^				
	Apical buds	Young stems	Upper leaves	Lower leaves	Roots
1	+ + + +	+ + + +	+ + + +	+ + + +	+ + + +
2	+ + + +	+ + + +	+ + + +	+ + + +	+ + + +
3	+ + + +	+ + + +	+ + + +	+ + + +	+ + + +
4	+ + + +	+ + + +	+ + + +	+ + + +	+ + + +
5	+ + + +	+ + + +	+ + + +	+ + + +	+ + + +

^a^*Symbols (+ + + +) indicate positive detection by RT-PCR for the four segments (RNA1, RNA2, RNA3, and RNA4) of TPNRBV. ^*b*^Tissues collected from different TPNRBV diseased tea plants were used as one biological replication*.

**Table 5:** Detection of TPNRBV via reverse-transcription (RT)-PCR in individual shoots or leaves of tea plant showing mixed symptoms of tea plant necrotic ring blotch disease.

Number of biological replications^b^	Shoots^a^		Leaves^a^	
	Upper leaves without symptoms	Lower leaves with symptoms	Leaf half with symptoms	Leaf half without symptoms
1	+ + + +	+ + + +	+ + + +	+ + + +
2	+ + + +	+ + + +	+ + + +	+ + + +
3	+ + + +	+ + + +	+ + + +	+ + + +
4	+ + + +	+ + + +	+ + + +	+ + + +
5	+ + + +	+ + + +	+ + + +	+ + + +
6	+ + + +	+ + + +	+ + + +	+ + + +
7	+ + + +	+ + + +	+ + + +	+ + + +
8	+ + + +	+ + + +	+ + + +	+ + + +
9	+ + + +	+ + + +	+ + + +	+ + + +
10	+ + + +	+ + + +	+ + + +	+ + + +
11	+ + + +	+ + + +	+ + + +	+ + + +
12	+ + + +	+ + + +	+ + + +	+ + + +
13	+ + + +	+ + + +	+ + + +	+ + + +
14	+ + + +	+ + + +	+ + + +	+ + + +
15	+ + + +	+ + + +	+ + + +	+ + + +

^a^*Symbols (++++) indicate positive detection by RT-PCR for the four segments (RNA1, RNA2, RNA3, and RNA4) of TPNRBV. ^*b*^Individual shoot/leaf from different TPNRBV diseased tea plant was used as one biological replication*.

## Discussion

The tea plant has a large geographic distribution in China. The differences in climatic conditions provide diverse ecological environments for tea plant growth and interactions with microorganisms. The 26 samples with discoloration symptoms were collected in Hangzhou. These samples are representative and common in tea-producing areas. In order to determine whether or not putative plant viruses are involved in discolored tea leaves, metagenomic sequencing was performed. NGS-based metagenomic analysis has been recently used to discover plant viruses (Adams et al., 2013, 2014). For the tea plant, the 102 Gbp of clean data generated by NGS indicates deep sequencing for virus identification. Potentially, due to the dilution effect of huge genome size (∼3.14 Gbp) of tea plant, no plant virus was discovered when we performed data analysis directly using the sequencing data without the removal of host genomic sequences (data not shown). Many viruses were identified only from the 2.82% of the data kept after carefully removing host genomic sequences, made possible by the completion of the tea plant whole-genome sequencing (Xia et al., 2017). These results suggest that deep sequencing and a proper data analysis strategy are crucial for virus discovery in the tea plant (Roossinck et al., 2015; Hadidi et al., 2016).

Metagenomic analysis is a powerful approach to investigate virus diversity in specific subjects. Meanwhile, virus taxonomy is being challenged by the explosion of information (Hadidi et al., 2016; Simmonds et al., 2017). However, NGS analysis does not generally provide the final word on a new virus, and further study is required to validate the existence of the detected viruses using multiple approaches (Hadidi et al., 2016). Only by homology annotation, in total 206 contigs from 36 plant virus species were identified as virus-associated sequences. After filter out false positive ones by removing the host genome insertion of virus related sequences and Sanger sequencing validation, seven plant viruses were identified. Except for APLPV and HJLV from genus *Ilarvirus* and BNRBV from genus *Blunervirus*, there are more tentative viruses identified in **Supplementary Material 3**, and many of them have not been assigned to genera. Most of the identified viruses have only been detected in few plant species or limited regions. Therefore, it is very difficult to identified plant viruses from tea plant without the application of NGS analysis. Based on genomic identification and molecular characterization, PnVA, MATV, and MATV2 were reported as tentative members of the genus *Totivirus*, family *Totiviridae* (Chen et al., 2016; Guo et al., 2016). PnVA is a double-stranded RNA (dsRNA) virus, first isolated from *Panax notoginseng* in Yunnan Province of China, and possibly associated with the mosaic disease (Guo et al., 2016). MATV is also a novel dsRNA virus from maize plant with a genome of 3,956 nucleotides in length, containing two ORFs with the potential to produce an ORF1–ORF2 fusion protein (Chen et al., 2016). A coat protein sequence of MATV2 (ANN85846.2) deposited in GenBank recently was reported as a virus associated to maize lethal disease in Ecuador. The contig TR42374| c0_g1_i1 has high homology to ANN85846.2, which can be speculated as an isolate of MATV. Hany et al. (2014) reported the first complete genome sequence of piper yellow mottle virus (PYMoV) and identified two distinct sequences tentatively named PDV1 and PDV2, respectively. PYMoV is a member of the family *Caulimoviridae*, genus *Badnavirus*, containing four ORFs. HJLV is a member of the genus *Ilarvirus*, isolated from 2 of 21 plants of *Humulus japonicus* grown from seed imported into the United Kingdom from China, and possesses a 26.3 kDa coat protein and four molecules of RNA (Scott and Zimmerman, 2006). However, the length of assembled contigs associated with these viruses in this study are still not long enough, and further complete genome sequence cloning is required to discover the specificity of identified viruses. Anyhow, the above information is significant to our understanding of the virosphere diversity in the tea plant. Our findings further demonstrate that the NGS deep sequencing approach is a feasible method for identification of plant viruses in field samples. However, one thing should be reminded of that, except for plant viruses, discoloration may be caused by other causal agents and even by abiotic effects, like variegation. Moreover, comprehensive pathological assays are required to establish correlation between the presence of the symptoms and the presence of the virus.

In this study, TPNRBV and TPLPV were the two most abundant plant viruses in the tea plant. TPNRBV and TPLPV had the closest sequence identities and evolutionary relationships with BNRBV and APLPV, respectively. However, the low sequence identity in nucleotide and amino acid levels between the viruses suggested that TPNRBV and TPLPV are two novel virus species from the tea plant. BNRBV was first discovered from highbush blueberries in several southeastern states of the United States by Quito-Avila et al. (2013). It caused irregularly shaped circular spots or blotches with green centers on the upper and lower surfaces of leaves. Later, a variant of BNRBV (BNRBV-RL) causing foliar red lesions was identified from highbush blueberries grown in Florida, United States (Cantu-Iris et al., 2013). BNRBV was placed into the new genus *Blunervirus* in the latest virus taxonomy released by ICTV in 2016 (Adams et al., 2016). To date, BNRBV has not been identified from other host plants, and no other virus species has been classified into this genus. Our genetic characterization indicated that TPNRBV has the same genome organization as BNRBV, containing four genomic segments (RNA1 to RNA4) and seven long ORFs (Quito-Avila et al., 2013). However, in the genome of BNRBV, a conserved sequence 5′-CACAAAT-3′ was found at the 5′ terminus of all the segments, while this sequence was only found in RNA1 of TPNRBV. No conserved sequence was found at 5′UTR among the segments of TPNRBV. Moreover, no poly(A) tail was found at either 3′ terminus of BNRBV RNA segment, while the presence of a poly(A) tail was validated in the four segments of TPNRBV, even though a high level of A-T content existed in the 3′ terminus of TPNRBV like that in BNRBV (Quito-Avila et al., 2013). This is a distinguishing feature between the two viruses. BNRBV causes necrotic ring blotch symptoms and red lesions (Cantu-Iris et al., 2013). Similarly, the symptoms caused by TPNRBV were variable, but the symptom of necrotic ring blotches on mature leaves was typical and commonly observed in different tea gardens and tea cultivars. Based on PCR analysis, specific primer pairs for each segment of TPBNRV were validated using the 26 employed samples and 15 samples randomly collected in the next year, which provide us an efficient approach to validate the existence of TPNRBV. Our study showed that the TPNRBV infected plants were still virus positive in the second year. Interestingly, detections on different tissues, shoots, and leaves with or without symptoms of tea plant necrotic ring blotch disease indicated a systemic movement of TPNRBV. Similar studies with BNRBV suggested that this virus infects only locally its host southern highbush blueberry (Robinson et al., 2016). BNRBV is not transmitted through vegetative propagation and will not persist in plants after natural defoliation in the fall. For TPNRBV, we tried to use back-inoculation to a healthy tea plant using injection or friction, in terms of Koch’s Postulates. Unfortunately, we have not observed typical symptomatic appearance in the inoculated plants so far. The usual long incubation period of woody plant viruses and putative high resistance of tea plant to viruses might take the responsibility to this result (Wang and Zhou, 2016). Obviously, more studies, including cutting propagation and grafting, are necessary to disclose the transmission and pathogenesis mechanism of TPNRBV, since the use of vegetative propagation is more popular in tea breeding. Also, once the tea plant is infected by TPNRBV, the disease spots will cover the entire leaves in lower portion of the canopy and lead to premature defoliation, which weaken the plants and reduce the output of fresh leaves/shoots significantly. Both genomic sequence and biological feature studies suggest that TPNRBV is a novel virus species of the *Blunervirus* genus distinguished with BNRBV. So far, the new genus *Blunervirus* has not been assigned to any family and there is no description on the shape of virus particle. Our first observation of TPNRBV in diseased tea leaves using TEM provides useful information for taxonomy and detection.

The viruses in the genus *Ilarvirus* (isometric labile ringspot viruses) have worldwide distribution and can infect herbaceous and woody hosts. In this genus, APLPV, ApMV, PDV, and PNRSV are invasive pathogens of many cultivated fruit trees, such as peach, cherry, prune, apple, and plum (Pallás et al., 2012). The complete genomic sequence of APLPV isolated from peach was first reported by Scott et al. (2001). Generally, ilarviruses can be classified into four subgroups, but APLPV is distinct from these subgroup members and is the least documented (Scott et al., 2001; Pallás et al., 2012). APLPV only has been reported in North America, Europe, the Mediterranean area, and most recently in Australia by deep sequencing (Paulsen and Fulton, 1968; Myrta et al., 2002; Alayasa et al., 2003; Kinoti et al., 2017). In this study, three long contigs that showed homology with segments of APLPV were obtained by metagenomic analysis based on NGS. Full-length genome cloning showed that this virus had three segments similar to those of ilarviruses and had the same genomic structure as APLPV. Nucleotide and amino acid identity analysis and phylogenetic analysis suggested that it is a novel virus from the tea plant, which we named TPLPV. Sequence analysis based on the corresponding movement and coat proteins of eight APLPV isolates from five Mediterranean countries indicated low genetic variability at the nucleotide and amino acid levels (up to 98%), regardless of their geographic origins (Herranz et al., 2008). RNA3 of TPLPV (TR32524| c6_g3_i1 in **Table 2**) had highest abundance and was annotated to putative movement protein 3a based on the best BLASTX hit (with a significant *E*-value of <1e^-5^). Actually, complete RNA3 of TPLPV encodes both movement and coat proteins which have only 24.8 and 31.7% identity, respectively, compared with those of APLPV. Our phylogenetic tree further showed that TPLPV was grouped in the same branch as APLPV, but distinct from known APLPV isolates. Particularly, the 3′ termini of members of family *Bromoviridae*, including viruses in genera *Alfamovirus*, *Anulavirus*, *Bromovirus*, *Cucumovirus*, *Ilarvirus*, and *Oleavirus*, are not polyadenylated, though generally are highly conserved within a species or isolates, and form strong secondary structures (reported by ICTV)^9^. However, a poly(A) tail was detected in the 3′ termini of the three segments of TPLPV. This is a unique property within ilarviruses or even family *Bromoviridae*. Further studies, including the validation of poly(A) tail both in TPNRBV and TPLPV using different strategies to eradicate the possible source of an experimental artifact and the dissection of poly(A) tail-based regulatory mechanism in virus replication, are still required in the future. Generally, the binding of coat protein to the 3′UTR of the genomic RNAs is required for ilarviruses in a phenomenon called genome activation to initiate infection (Pallás et al., 2012). In PNRSV, the RBD in N-terminus and DR in C-terminus of coat protein are essential in the binding process (Aparicio et al., 2003). A high proportion of R residues are found in TPLPV RBD like as other ilarviruses coat proteins, which are responsible for the binding to 3′UTR (Sánchez-Navarro and Pallás, 1997; Aparicio et al., 2003). Alignment of the coat protein of TPLPV and other ilarviruses showed that the residues in RBD region were not highly conserved except a R residue in aa 23 (**Figure 5E**), which supports the observation that a central R residue in consensus sequence (Q/K/R-P/N-T-X-**R**-S-R/Q-Q/N/S-W/F/Y-A) of RBD is essential for the binding to the RNA (Ansel-McKinney et al., 1996). On the contrary, the DR in C-terminus of coat protein was highly conserved, which plays important role in coat protein dimerization in the genome activation process (Aparicio et al., 2006). It is assumable that TPLPV may have same genome activation process with known ilarviruses through the binding of coat protein to 3′UTR of the genomic RNAs. Corresponding to the binding feature of coat protein, the 3′UTR of ilarviruses commonly form several stem-loop structures flanked by non-paired sequences (Pallás et al., 1999). The 3′UTR of PNRSV RNA3 has been validated the formation of conformational switch between stem-loop and pseudoknot conformers, which can be triggered by coat protein and is recognized as an important regulatory mechanism in the life cycle of most ilarviruses (Aparicio et al., 2003). RNA secondary structure prediction showed that the 3′UTR of TPLPV RNA3 had very similar composition of stem-loop structures with PNRSV. Likewise, the relative high sequence conservation of RNA3 3′UTR between TPLPV and related ilarviruses supports our assumption. Of course, *in vitro* and *in vivo* evidences are required. Currently, few viruses having a close evolutionary relationship with APLPV have been discovered, and their full-length genomic sequences are mostly unknown. The discovery of TPLPV and its genome sequence will provide valuable information for better understanding the genetic diversity of ilarviruses. Moreover, PCR-validated specific primer pairs for each segment of TPLPV were designed for fast virus identification. Among the 26 samples, four were TPLPV-infected. Unluckily, taking the positive samples in 2016 as control, no TPLPV-positive samples were detected in the 15 samples randomly collected in the second year. These data indicate that TPLPV may be cultivar-specific or season-specific. Also, the possible particularity of its transmitting vector or uneven distribution in host may cause the negative detection. Therefore, intensive sampling in the TPLPV positive fields and comprehensive biological feature study are necessary.

Tea plant is an economically important woody species having an over 30 year’s active producing period under conventional cultivation and management. In comparison of annual or biennial crops, systemic diseases especially caused by viruses will cause more serious losses to tea production and bring challenges to disease management. Therefore, virus identification and determining the resistance mechanisms of the tea plant to viruses are meaningful to tea plant disease management and will fill valuable gaps in the knowledge of tea plant virology. Moreover, virus identification and characterization have great potential value for developing virus-induced gene silencing (VIGS) vector systems for the tea plant because stable genetic transformation to downregulate gene expression is still mostly not achievable (Lange et al., 2013; Li M. et al., 2015). It can be expected that tea plant viruses will become an important tool for functional identification and gene manipulation in the genetic breeding of the tea plant (Zaidi and Mansoor, 2017).

## Conclusion

Taking advantage of NGS, metagenomic analysis was performed to investigate plant viruses in tea plants. Ultimately, seven kinds of plant viruses were identified on the basis of homology annotation and Sanger sequencing. TPNRBV and TPLPV were the two most abundant plant viruses in our tea plant samples. This first report of these two tentative novel viruses includes full-length genome sequence cloning and genetic characterization. Moreover, specific primer pairs for PCR analysis were designed and validated for TPNRBV and TPLPV identification. Sequence conservation analysis and RNA secondary structure prediction assumed that the genome expression of TPLPV may be activated by the binding of coat protein to 3′UTR of the genomic RNAs. RT-PCR detections of TPNRBV in the symptomatic and asymptomatic tissues from field plants showing tea plant necrotic ring blotch disease indicate that TPNRBV can infect tea plants systemically. Particularly, the suspected virus particles of TPNRBV were observed using TEM. Our results advance our understanding of tea plant virology and will be important for the genetic breeding of tea plants in the future.

## Author Contributions

XH, YY, and XW conceived and designed the experiments. XH, WZ, YL, WQ, and LW performed the experiments. XH, FZ, and JZ analyzed the data. XH, WQ, and YW contributed with plant materials. XH wrote the paper.

## Conflict of Interest Statement

The authors declare that the research was conducted in the absence of any commercial or financial relationships that could be construed as a potential conflict of interest.

## Footnotes

1 https://github.com/najoshi/sickle

2 https://sourceforge.net/projects/bbmap/

3 https://github.com/trinityrnaseq/trinityrnaseq

4 http://www.cyverse.org/discovery-environment

5 ftp://ftp.ncbi.nlm.nih.gov/blast/db/

6 https://www.ncbi.nlm.nih.gov/tools/primer-blast/

7 https://www.ebi.ac.uk/Tools/psa/

8 http://rna.tbi.univie.ac.at/cgi-bin/RNAWebSuite/RNAcofold.cgi

9 https://talk.ictvonline.org/ictv-reports/
