# Supplementary material for: Discovery of Plant Viruses From Tea Plant (Camellia sinensis (L.) O. Kuntze) by Metagenomic Sequencing
Source: Front Microbiol. 2018 Sep 11;9:2175. doi: 10.3389/fmicb.2018.02175 (PMC6141721; doi:10.3389/fmicb.2018.02175)

**Supplementary Material 1.** The viruses validated by Sanger sequencing of the most-abundant contig

| **Virus name** | **Most abundant contig ID** | **Contig length** | **Log2 of FPKM** | **Primer information (5’ to 3’)** | **Amplicon length** | **Annealing temperature** | **Number in gel^a^** |
| --- | --- | --- | --- | --- | --- | --- | --- |
| American plum line pattern virus | TR32524\|c6_g3_i1 | 2136 bp | 16.63 | Forward: GGGCACTAGGGACAGACCTA  Reverse: GCGACGAAAGAAGCCGTTAC | 439 bp | 55ºC | 1 |
| cacao mild mosaic virus | TR386\|c0_g1_i1 | 243 bp | 1.00 | Forward: TACATCACGAGGAGTAAGGGC  Reverse: AAACTGGTTCCGCATGTTGAG | 200 bp | 55ºC | 2 |
| maize-associated totivirus 2 | TR42374\|c0_g1_i1 | 347 bp | 2.81 | Forward: AAATTTTGGGCATACATACAC  Reverse: TCAACGTCGGAGAACAAG | 293 bp | 53ºC | 3 |
| piper dna virus 2 | TR45179\|c0_g1_i1 | 375 bp | 1.58 | Forward: TGATGGATGGACCCCTCGAA  Reverse: CTGGCCAGAGTTGTTGAGGAT | 225 bp | 55ºC | 4 |
| blueberry necrotic ring blotch virus | TR46240\|c4_g1_i1 | 5938 bp | 17.09 | Forward: GTGTTGAACCACCAAGTCGC  Reverse: CTTGCACACGTTTGGGTCAG | 490 bp | 55ºC | 5 |
| sweet potato badnavirus a | TR46271\|c2_g7_i2 | 258 bp | 1.58 | Forward: ACCACTATCTACCACCAACACC  Reverse: CAGAGATCTCGAAAGACTGGCA | 206 bp | 55ºC | 6 |
| Cmmelina yellow mottle virus | TR46271\|c3_g4_i1 | 517 bp | 4.09 | Forward: GGTGTCGGGTACTAGCTTGG  Reverse: CGTCGCTGGTTCTGACGATA | 229 bp | 55ºC | 7 |
| sweet potato badnavirus b | TR46271\|c3_g6_i4 | 631 bp | 4.55 | Forward: GAATGACCAACCGAGGTGGA  Reverse: CTGGCTTGGTCAATGGCATC | 289 bp | 55ºC | 8 |
| grapevine roditis leaf discoloration-associated virus | TR46309\|c1_g5_i2 | 511 bp | 4.95 | Forward: TCTCCTGGGATCGCTTTGAC  Reverse: TGGACGGGAGATCAAGCAAC | 293 bp | 55ºC | 9 |
| piper yellow mottle virus | TR46309\|c1_g8_i7 | 1094 bp | 4.45 | Forward: CGATGTTGGCGCAGGAGTAAT  Reverse: AGGCTGCTTCTGTTGTCTTCTTTC | 448 bp | 53ºC | 10 |
| Coccinia mosaic Tamil Nadu virus | TR52511\|c1_g1_i1 | 971 bp | 4.00 | Forward: ATCTCTCTGCCTCTGCGGTA  Reverse: AGGAAAAACCGCCTAAGCCA | 638 bp | 55ºC | 11 |
| maize associated totivirus | TR52544\|c0_g1_i1 | 407 bp | 1.37 | Forward: CATGTCCCTTTCGAATTCCGC  Reverse: TGGGGAAAACAGCGAGCAAT | 267 bp | 55ºC | 12 |
| Dioscorea bacilliform AL virus | TR61680\|c0_g6_i1 | 503 bp | 2.32 | Forward: GGTATTCGGAAGGCAACAACG  Reverse: ATCAACACCTTCGGACACCG | 273 bp | 55ºC | 13 |
| banana streak virus | TR61680\|c0_g7_i1 | 523 bp | 3.46 | Forward: GCCTCGATTTCCCGGATCAA  Reverse: TGCAAAGCACATCCAGTCCA | 345 bp | 55ºC | 14 |
| Dioscorea rotundata virus | TR61708\|c2_g2_i2 | 1006 bp | 4.32 | Forward: GATGTCTGCTCTTGCTGGGT  Reverse: TCACAAACGGAGCCCTCAAA | 445 bp | 55ºC | 15 |
| cacao swollen shoot virus | TR61708\|c3_g9_i2 | 528 bp | 3.46 | Forward: CCGTTCGTCGTTGGAACTCT  Reverse: CGGGGAGTCAAAGCCATACC | 367 bp | 55ºC | 16 |
| Humulus japonicus latent virus | TR63699\|c0_g1_i1 | 226 bp | 1.00 | Forward: GACTCTGAATCATCTTTTTAT  Reverse: CGCTGTCGGTGTTTGGTA | 146 bp | 53ºC | 17 |
| watermelon chlorotic stunt virus-[sd] | TR67857\|c0_g1_i3 | 1137 bp | 5.86 | Reverse: AAGCGACGGGTTGTAACTGT | 272 bp | 55ºC | 18 |
| Panax notoginseng virus a | TR73446\|c0_g2_i1 | 1657 bp | 5.16 | Forward: GTCAATCTCCACCCCGACAG  Reverse: ATCGAGATGGGAATGGGCAC | 576 bp | 55ºC | 19 |
| dioscorea bacilliform virus | TR75257\|c1_g8_i1 | 454 bp | 3.54 | Forward: ATTGGCGTCGTTCCTCGAAT  Reverse: CGAACACAGCTACCCTCTTGA | 325 bp | 55ºC | 20 |
| taro bacilliform virus | TR75257\|c1_g11_i2 | 311 bp | 0.00 | Forward: TCCAGTGAAACCCTGTTGTGA  Reverse: TCAAGGTAGGTTAGGCGTGA | 229 bp | 55ºC | 21 |
| fig badnavirus 1 | TR80150\|c0_g1_i1 | 510 bp | 5.36 | Forward: TTCGTCGCCTTTGTGCCTTA  Reverse: GGCTTCTCCACCTTGCCAAT | 291 bp | 55ºC | 22 |
| fowl aviadenovirus 1 | TR85195\|c0_g2_i1 | 316 bp | 0.00 | Forward: AGCGATTCTCCCAAAGAGCC  Reverse: AGGCAAAACTCTGTCGAACAT | 230 bp | 55ºC | 23 |
| Citrus chlorotic dwarf associated virus | TR88903\|c0_g1_i5 | 539 bp | 3.83 | Forward: AATTGTGTCGTCCGAGGCTT  Reverse: ACGAAGAAGGACGAACGAGG | 201 bp | 55ºC | 24 |
| hibiscus bacilliform virus gd1 | TR91278\|c0_g1_i3 | 523 bp | 3.57 | Forward: AGGCATCCTTTCCTCATCCT  Reverse: ACCCTTGCATAGCCACATGAA | 311 bp | 55ºC | 25 |
| piper dna virus 1 | TR91406\|c2_g1_i2 | 718 bp | 2.58 | Forward: AACAACCCCGCTAAACGACT  Reverse: ATGGTTGACCGCATCTTGCT | 305 bp | 55ºC | 26 |
| cacao swollen shoot cd virus | TR91411\|c3_g4_i1 | 387 bp | 2.58 | Forward: CTTGGAGAAGCAGGCGGATT  Reverse: GGCAAGAGTCTCCTGTCGAT | 245 bp | 55ºC | 27 |
| sugarcane bacilliform virus | TR91465\|c2_g3_i1 | 296 bp | 1.58 | Forward: ATCCATCTGGGCTTTGGCAAT  Reverse: GCTCCAGCAGTCTTTCAGAG | 204 bp | 55ºC | 28 |
| taro bacilliform ch virus | TR91465\|c2_g9_i1 | 474 bp | 3.92 | Forward: TAAGGGGGCGTCCTTCTACA  Reverse: TTGCCGGATCAGGGTTTTCA | 391 bp | 55ºC | 29 |

^a^ PCR products in 2% gel were showed in below picture. The corresponding number of each virus was marked on the top of lane. Same DNA marker was used in gel electrophoresis.
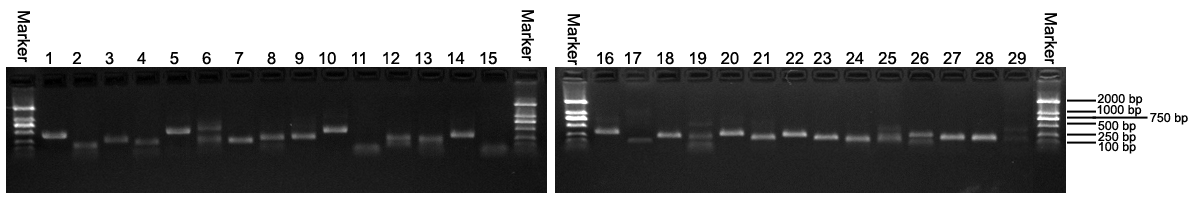

Supplement: Supplementary file 1 [file Data_Sheet_1.DOCX]
