# Supplementary material for: Discovery of Plant Viruses From Tea Plant (Camellia sinensis (L.) O. Kuntze) by Metagenomic Sequencing
Source: Front Microbiol. 2018 Sep 11;9:2175. doi: 10.3389/fmicb.2018.02175 (PMC6141721; doi:10.3389/fmicb.2018.02175)
Supplement: Supplementary file 2 [file Data_Sheet_2.DOCX]

**Supplementary Material 2.** Primer information for full length sequence cloning of tea plant line pattern virus (TPLPV) and tea plant necrotic ring blotch virus (TPNRBV)

| Cloning type | Virus segment | Primer name | Sequence (5’ to 3’) | Optimal annealing temperature |
| --- | --- | --- | --- | --- |
| Middle fragment cloning | TPNRBV RNA1 | BNR1-FL5190-F | GGCCCTGACAACGCAAAAGAACTG | 58ºC |
|  |  | BNR1-FL5190-R | GCTGTCACTCCCAAACGCTCCATC |  |
|  | TPNRBV RNA2 | BNR2-FL3588-F | CAATCATACGTGCGGTCAGTCAGG | 57ºC |
|  |  | BNR2-FL3588-R | ATGGCCAAAATAGCGAACACAGAAT |  |
|  | TPNRBV RNA3 | BNR3-FL2259-F | CCGCGTCGTTCTCTGGTCTAATAC | 56ºC |
|  |  | BNR3-FL2259-R | CCGGGTGTGGTGTTGCTGTC |  |
|  | TPNRBV RNA4 | BNR4-FL858-F | CGTGTCTACCGTTACCGTCTCTGC | 57ºC |
|  |  | BNR4-FL858-R | TCTGATAATCGCGCCACTGAACC |  |
|  | TPLPV RNA1 | ARP1-FL2555-F | GCAGATTAAGAAGACGGTCGCAACTA | 56ºC |
|  |  | ARP1-FL2555-R | AAACGCACTGGAACAACAACACTCAA |  |
|  | TPLPV RNA2 | ARP3-FL1807-F | ACGTTTACAACTGGCTGCGGTAGAG | 57ºC |
|  |  | ARP3-FL1807-R | CGAATCCCCATAGGTTCATCTCGTA |  |
| RACE cloning | TPLPV RNA1 | APR1-5’race | TCAGGAGTCATCATGATAGTTGCGACCGTCTTC | 68ºC |
|  |  | APR1-3’race | TCACTTGAAAGGGAGAAGGGTGCTTACTGTGCA | 68ºC |
|  | TPLPV RNA2 | APR2-5’race | GGTGTTCTCTTCGACCTTATCAATCGGCATATCAT | 68ºC |
|  |  | APR2-3’race | GATGGTCGTGGAAAAGTGGTCGCTGTCCCA | 68ºC |
|  | TPLPV RNA3 | APR3-5’race | GCGATAACCGTTGGTAAAAACACAAGCAGGAA | 68ºC |
|  |  | APR3-3’race | AGTGAAGACGTTGTGATGTGTCGACTCAGAAGTGA | 68ºC |
|  | TPNRBV RNA1 | BNR1-5’race | CGAACCCAGTGACAATCGTGAGGTCCCAACTCT | 68ºC |
|  |  | BNR1-3’race | TCCTACCATACGTCATGAACCCGTTGTGGATGTG | 68ºC |
|  | TPNRBV RNA2 | BNR2-5’race | GTGAACGATGCAGACAACAAACATTTATTTCGCAA | 68ºC |
|  |  | BNR2-3’race | TCTCAGGGCTTGTTGGTTTTGTTAATCGAATGC | 68ºC |
|  | TPNRBV RNA3 | BNR3-5’race | CCAATGAAAACAACAACGCAAGTCAACAAATCTCT | 68ºC |
|  |  | BNR3-3’race | AACTTGCCTAATCTTATGGTCGATGTTGCGCAT | 68ºC |
|  | TPNRBV RNA4 | BNR4-5’race | TGGATGAAAAATGACTATCTGAGGTCCCTCCGATG | 68ºC |
|  |  | BNR4-3’race | GAAAGTCGGTGGGCTCACGCCTCAGATAACGT | 68ºC |
