# Supplementary material for: Discovery of Plant Viruses From Tea Plant (Camellia sinensis (L.) O. Kuntze) by Metagenomic Sequencing
Source: Front Microbiol. 2018 Sep 11;9:2175. doi: 10.3389/fmicb.2018.02175 (PMC6141721; doi:10.3389/fmicb.2018.02175)
Supplement: Supplementary file 4 [file Data_Sheet_4.DOCX]

**Supplementary Material 4.** PCR detection to validate the assembly contigs annotated as American plum line pattern virus (APLPV) and blueberry necrotic ring blotch virus (BNRBV).


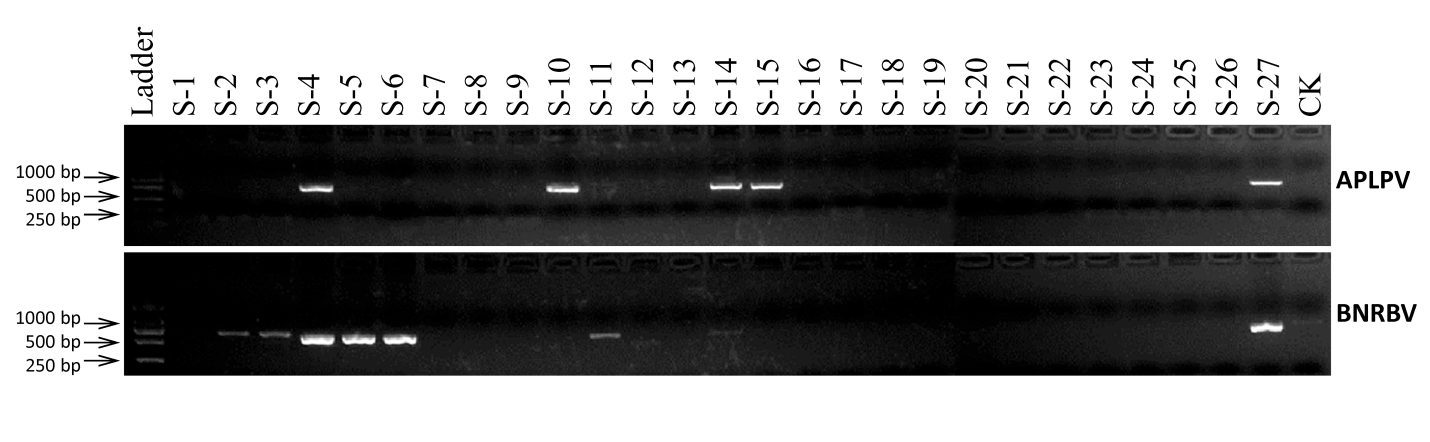


Figure 1 Gel picture of the PCR validation

Note: S-1 to S-26 stands for the cDNA templates from 26 typical albino and chlorina leaves of tea plants (*Camellia sinensis* (L.) O. Kuntze) employed in this study. S-27 is the cDNA template reverse transcribed from RNA mixture of 26 samples used for RNA-Seq library construction. CK is the template from normal green leaves of tea cultivar.

Table 1 The contig and primer information used for above PCR validation

| Detected contig | Primer name | Sequence (5’ to 3’) | Amplicon size | Optimal Tm |
| --- | --- | --- | --- | --- |
| TR32524\|c6_g3_i1 | APR-F1 | TGGTCATGTTGCGAATGTTT | 561 bp | 52℃ |
|  | APR-R1 | GCGTCTGTTTCGGGTATGAT |  |  |
| TR46240\|c4_g1_i1 | BNR-F1 | ATTGTCACTGGGTTCGCTATG | 615 bp | 52℃ |
|  | BNR-R1 | ACGAATTTATCCGCCTCACTT |  |  |
