# Supplementary material for: Discovery of Plant Viruses From Tea Plant (Camellia sinensis (L.) O. Kuntze) by Metagenomic Sequencing
Source: Front Microbiol. 2018 Sep 11;9:2175. doi: 10.3389/fmicb.2018.02175 (PMC6141721; doi:10.3389/fmicb.2018.02175)
Supplement: Supplementary file 5 [file Data_Sheet_5.DOCX]

**Supplementary Material 5.** Identity and similarity analyses of the full length sequences of tea plant line pattern virus (TPLPV) and tea plant necrotic ring blotch virus (TPNRBV).

Table 1. Identity and similarity analyses of the full-length sequence of blueberry necrotic ring blotch virus (BNRBV) and tea plant necrotic ring blotch virus (TPNRBV)

| **Segment** | **Coding protein** | **At the nucleotide level** | | | | | | | |  | **At the amino acid level** | | | | | |
| --- | --- | --- | --- | --- | --- | --- | --- | --- | --- | --- | --- | --- | --- | --- | --- | --- |
|  |  | **BNRBV** | |  |  | | **TPNRBV** | | **Identity (%)** |  | **BNRBV** | |  | **TPNRBV** | **Identity (%)** | **Similarity (%)** |
|  |  | **Accession number** | **Segment length (bp)** |  | **Accession number** | | | **Segment length (bp)** |  |  | **Accession number** | **protein length (bp)** |  | **protein length (bp)** |  |  |
| RNA1 | Methyltransferase helicase | JN651148 | 5906 |  |  | MG781152 | | 5922 | 44.2 |  | AEQ55299 | 1887 |  | 1861 | 22.8 | 37.3 |
| RNA2 | Polymerase | JN651149 | 3935 |  |  | MG781153 | | 4107 | 47.7 |  | AEQ55300 | 1144 |  | 1211 | 31.3 | 48.2 |
| RNA3 | Hypothetical protein | JN651150 | 2588 |  |  | MG781154 | | 2678 | 44.6 |  | AEQ55301 | 59 |  | 123 | 10.6 | 16.3 |
|  | Hypothetical protein |  |  |  |  |  |  |  |  |  | AEQ55302 | 256 |  | 250 | 23.7 | 38.0 |
|  | p24 |  |  |  |  |  |  |  |  |  | AEQ55303 | 202 |  | 195 | 26.5 | 42.6 |
|  | Hypothetical protein |  |  |  |  |  |  |  |  |  | AEQ55304 | 259 |  | 210 | 25.5 | 37.4 |
| RNA4 | Movement protein | JN651151 | 1724 |  |  | MG781155 | | 4107 | 42.4 |  | AEQ55305 | 311 |  | 315 | 37.3 | 55.3 |

Table 2. Identity and similarity analyses of the full-length sequence of American plum line pattern virus (APLPV) and tea plant line pattern virus (TPLPV)

| **Segment** | **Coding protein** | **At the nucleotide level** | | | | | |  | **At the amino acid level** | | | | | | | |
| --- | --- | --- | --- | --- | --- | --- | --- | --- | --- | --- | --- | --- | --- | --- | --- | --- |
|  |  | **APLPV** | |  | **TPLPV** | | **Identity (%)** |  |  | **APLPV** | |  |  | **TPLPV** | **Identity (%)** | **Similarity (%)** |
|  |  | **Accession number** | **Segment length (bp)** |  | **Accession**  **number** | **Segment length (bp)** |  |  |  | **Accession number** | **protein length (bp)** |  |  | **protein length (bp)** |  |  |
| RNA1 | Replicase p1 | AF235033 | 3373 |  | MG781156 | 3373 | 53.8 |  |  | AAK15026 | 1062 |  |  | 1042 | 42.0 | 59.4 |
| RNA2 | Polymerase p2 | AF235165 | 2404 |  | MG781157 | 2354 | 51.7 |  |  | AAK15028 | 740 |  |  | 696 | 41.8 | 56.9 |
| RNA3 | Movement protein 3a | AF235166 | 2053 |  | MG781158 | 2142 | 44.1 |  |  | AAK15029 | 290 |  |  | 289 | 24.8 | 37.9 |
|  | Coat protein |  |  |  |  |  |  |  |  | AAK15030 | 216 |  |  | 203 | 31.7 | 46.4 |
