# Supplementary material for: Discovery of Plant Viruses From Tea Plant (Camellia sinensis (L.) O. Kuntze) by Metagenomic Sequencing
Source: Front Microbiol. 2018 Sep 11;9:2175. doi: 10.3389/fmicb.2018.02175 (PMC6141721; doi:10.3389/fmicb.2018.02175)
Supplement: Supplementary file 6 [file Data_Sheet_6.docx]

**
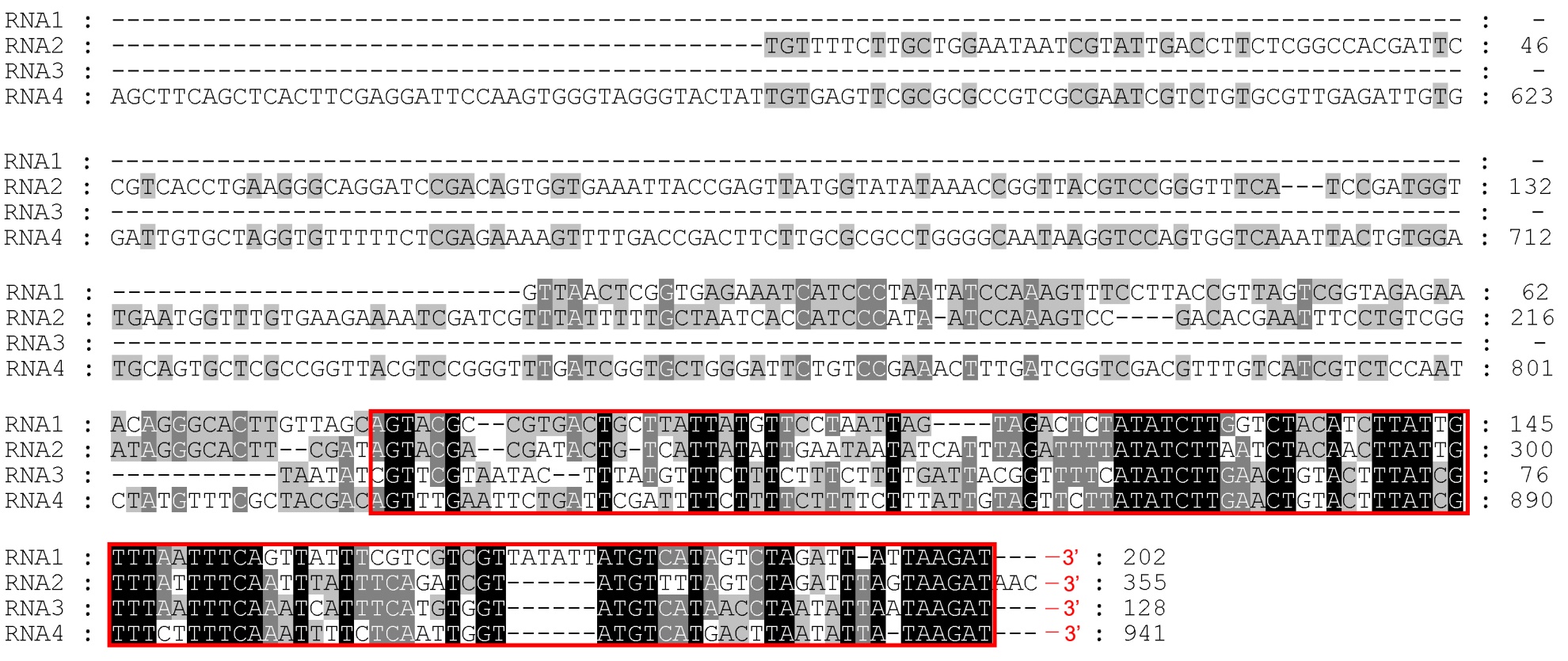
Supplementary Material 6.**  Alignment of 3’ non-coding regions of tea plant necrotic ring blotch virus (TPNRBV). Partial sequences of four segments (RNA1 to RNA4) of TPNRBV from 3’ terminus were displayed. Black shaded areas indicate nucleotides conserved across the four segments. Red boxes indicate the highly conserved regions.
