# Supplementary material for: Discovery of Plant Viruses From Tea Plant (Camellia sinensis (L.) O. Kuntze) by Metagenomic Sequencing
Source: Front Microbiol. 2018 Sep 11;9:2175. doi: 10.3389/fmicb.2018.02175 (PMC6141721; doi:10.3389/fmicb.2018.02175)
Supplement: Supplementary file 7 [file Data_Sheet_7.docx]

**Supplementary Material 7.** Gel electrophoresis of the reverse-transcription (RT)-PCR productions in detection of tea plant necrotic ring blotch virus.


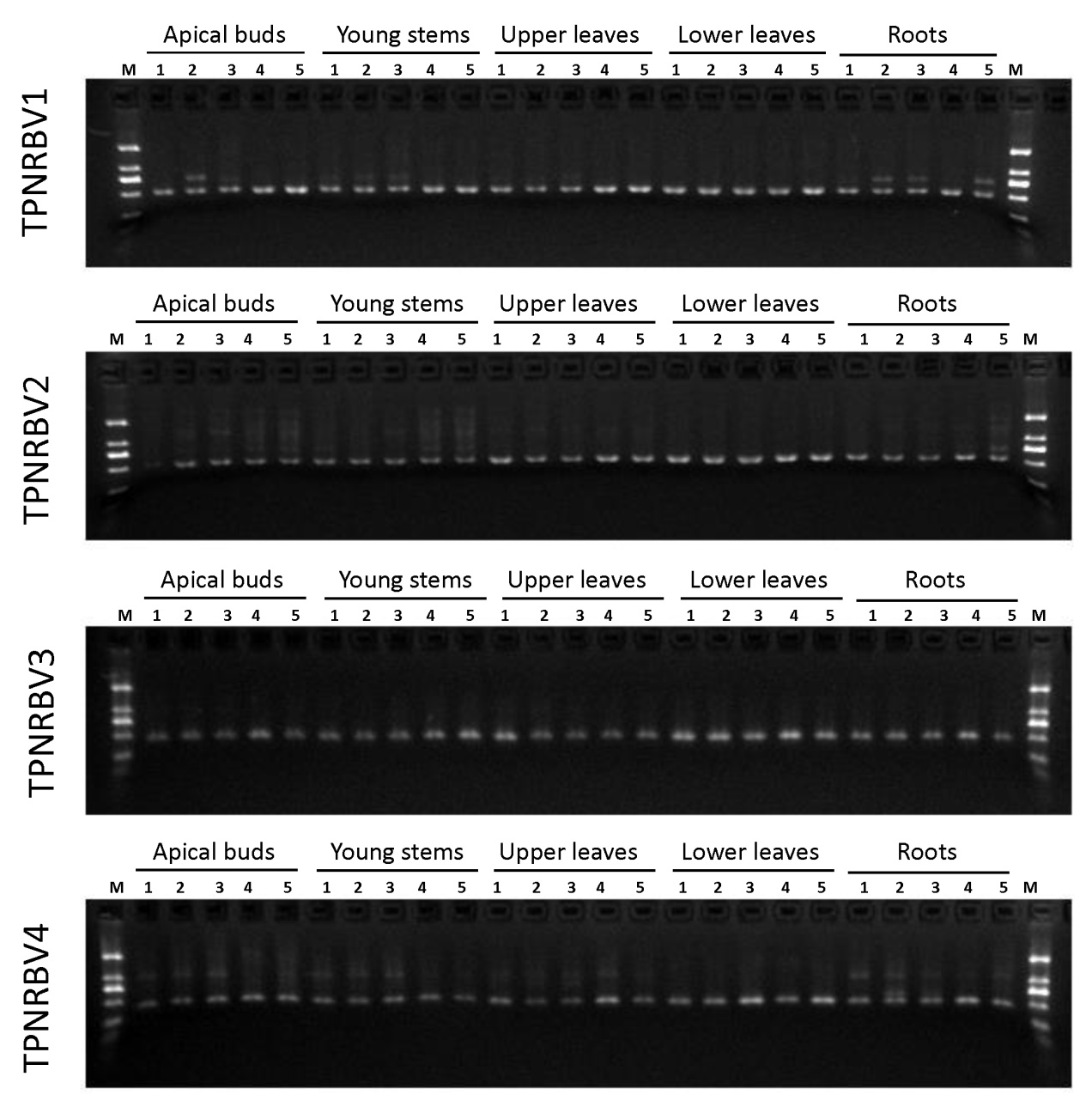
 Supplementary Material 7-1. Electrophoresis detection of tea plant necrotic ring blotch virus (TPNRBV) via reverse-transcription (RT)-PCR in different tissues of tea plant showing mixed symptoms of tea plant necrotic ring blotch disease. Five biological replicates (1 to 5) were performed for each tissue collection. Specific primer pairs TPNRBV1, TPNRBV2, TPNRBV3 and TPNRBV4 for segment RNA1, RNA2, RNA3 and RNA4 of TPNRBV detection were employed in RT-PCR independently. The DNA marker (M) information was provided in S7-4.


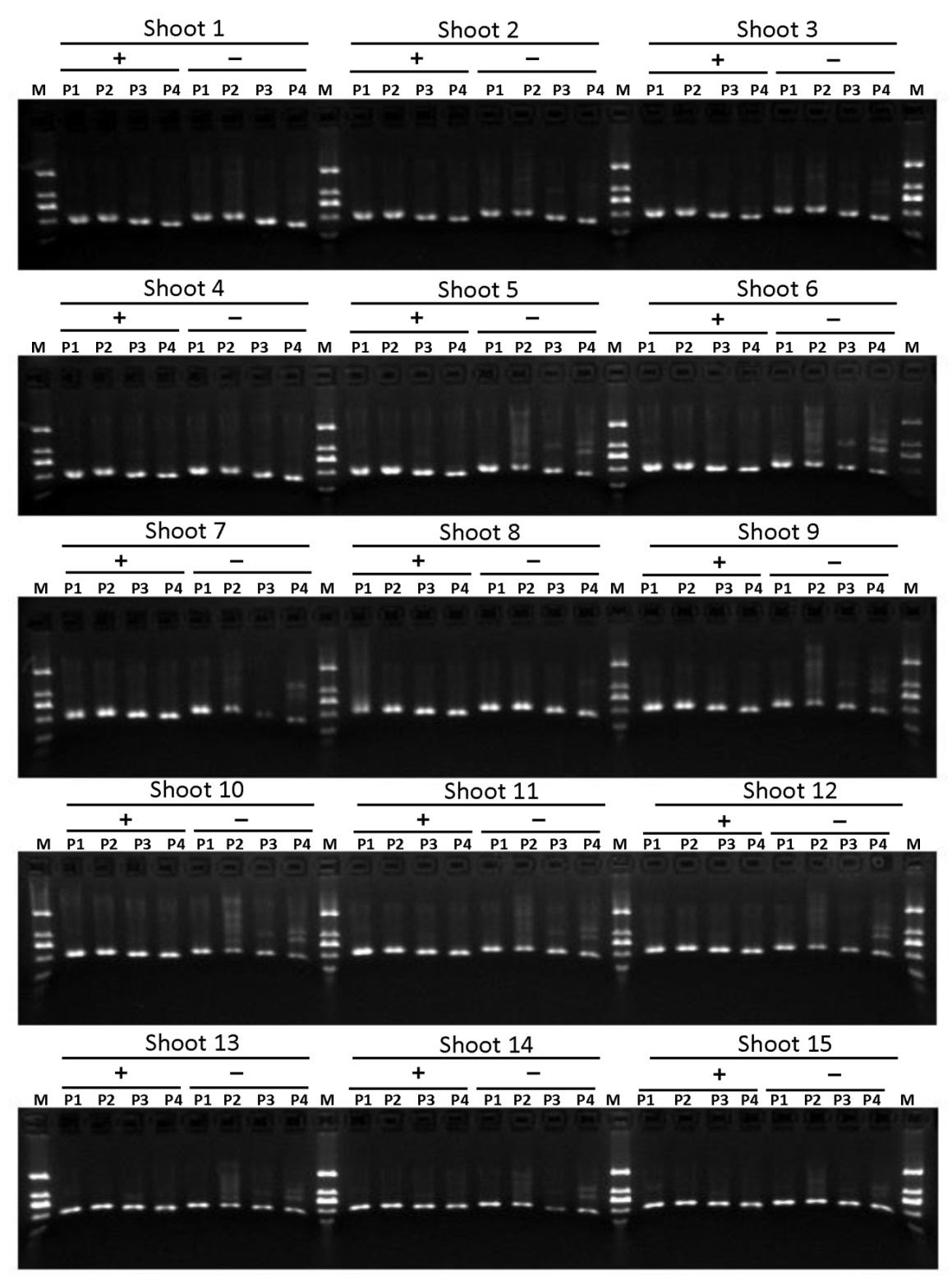
 Supplementary Material 7-2. Electrophoresis detection of tea plant necrotic ring blotch virus (TPNRBV) via reverse-transcription (RT)-PCR in individual shoots (shoot 1 to shoot 15) from different tea plants showing mixed symptoms of tea plant necrotic ring blotch disease. Symbols indicate upper leaves without symptoms (–) and lower leaves with symptoms (+) in one shoot. P1 to P4 indicate the four specific primer pairs for segment RNA1, RNA2, RNA3 and RNA4 of TPNRBV respectively. The DNA marker (M) information was provided in S7-4.


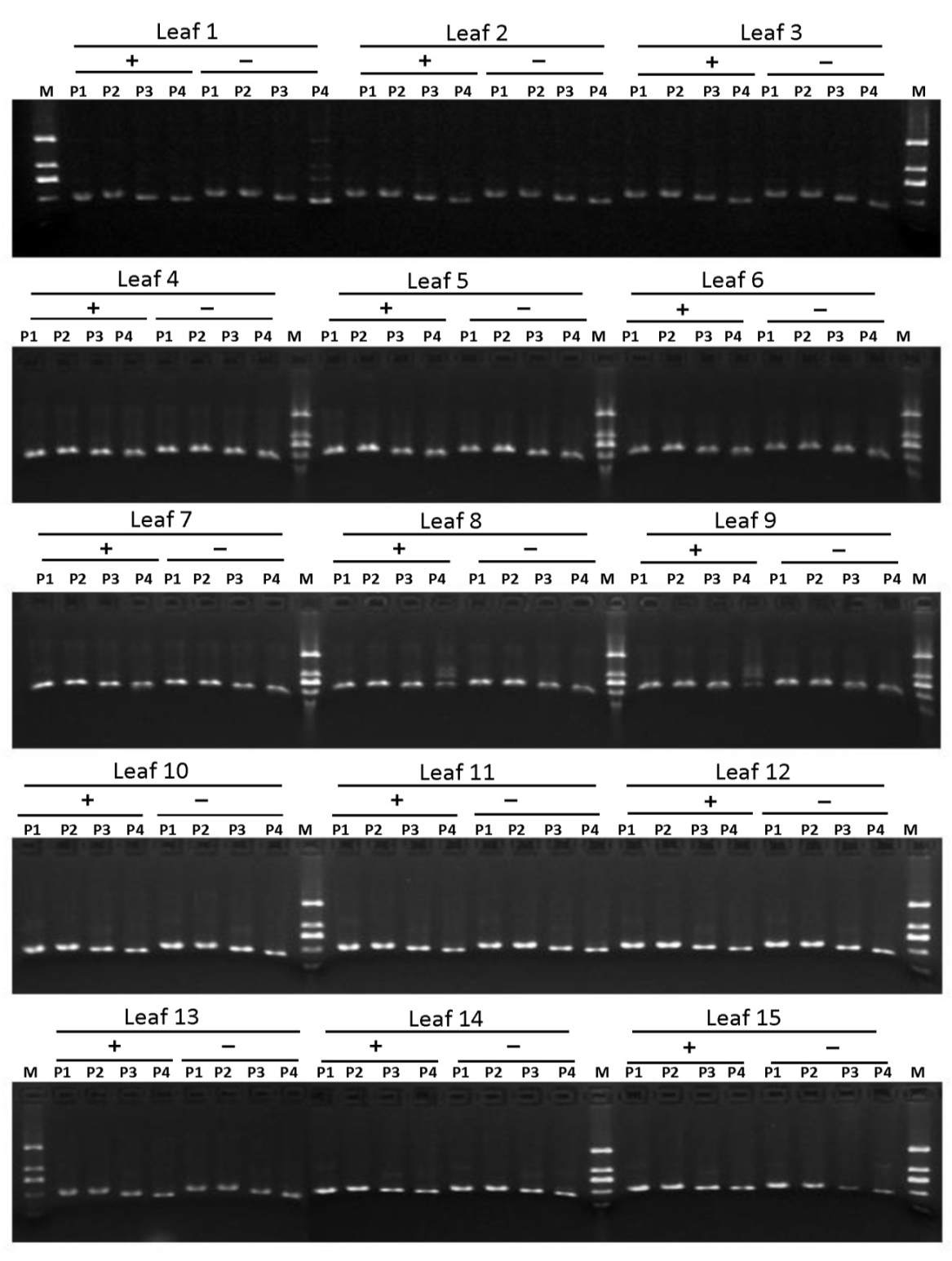
 Supplementary Material 7-3. Electrophoresis detection of tea plant necrotic ring blotch virus (TPNRBV) via reverse-transcription (RT)-PCR in individual leaves (leaf 1 to leaf 15) from different tea plants showing mixed symptoms of tea plant necrotic ring blotch disease. Symbols indicate leaf half without symptoms (–) and leaf half with symptoms (+) in one leaf. P1 to P4 indicate the four specific primer pairs for segment RNA1, RNA2, RNA3 and RNA4 of TPNRBV respectively. The DNA marker (M) information was provided in S7-4.


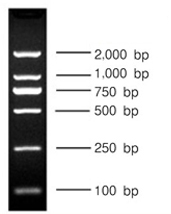
 Supplementary Material 7-4. The size of each band of DNA Marker (M) in 3% gel electrophoresis. Same marker was used in above gel analyses.
